# Genome Mining and Gene Expression Reveal Maytansine Biosynthetic Genes from Endophytic Communities Living inside *Gymnosporia heterophylla* (Eckl. and Zeyh.) Loes. and the Relationship with the Plant Biosynthetic Gene, Friedelin Synthase

**DOI:** 10.3390/plants11030321

**Published:** 2022-01-25

**Authors:** Thanet Pitakbut, Michael Spiteller, Oliver Kayser

**Affiliations:** 1 Technical Biochemistry, Department of Biochemical and Chemical Engineering, TU Dortmund University, 44227 Dortmund, Germany; thanet.pitakbut@tu-dortmund.de; 2 Department of Chemistry and Chemical Biology, Institute of Environmental Research (INFU), TU Dortmund University, 44227 Dortmund, Germany; michael.spiteller@tu-dortmund.de

**Keywords:** AHBA synthase gene, halogenase gene, FRS gene, maytansine-producible endophytes, endophyte–endophyte interaction, plant–endophyte interaction

## Abstract

Even though maytansine was first discovered from Celastraceae plants, it was later proven to be an endophytic bacterial metabolite. However, a pure bacterial culture cannot synthesize maytansine. Therefore, an exclusive interaction between plant and endophytes is required for maytansine production. Unfortunately, our understanding of plant–endophyte interaction is minimal, and critical questions remain. For example: how do endophytes synthesize maytansine inside their plant host, and what is the impact of maytansine production in plant secondary metabolites? Our study aimed to address these questions. We selected *Gymnosporia heterophylla* as our model and used amino-hydroxybenzoic acid (AHBA) synthase and halogenase genes as biomarkers, as these two genes respond to biosynthesize maytansine. As a result, we found a consortium of seven endophytes involved in maytansine production in *G. heterophylla*, based on genome mining and gene expression experiments. Subsequently, we evaluated the friedelin synthase (FRS) gene’s expression level in response to biosynthesized 20-hydroxymaytenin in the plant. We found that the FRS expression level was elevated and linked with the expression of the maytansine biosynthetic genes. Thus, we achieved our goals and provided new evidence on endophyte–endophyte and plant–endophyte interactions, focusing on maytansine production and its impact on plant metabolite biosynthesis in *G. heterophylla*.

## 1. Introduction

**Figure 1:**
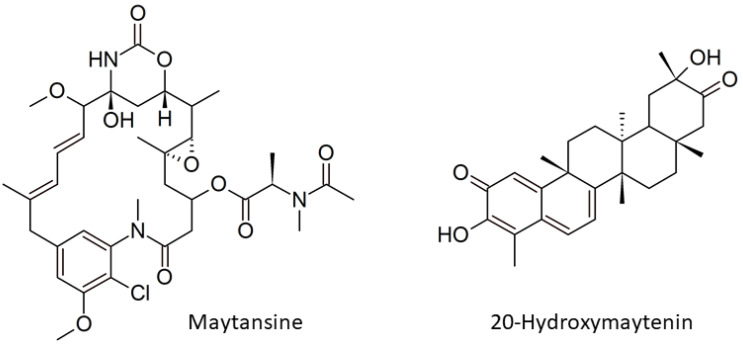
Chemical structure of maytansine (**left**) and 20-hydroxymaytenin (**right**).

Endophytism defines a unique relationship between symbiosis microbes colonized in their plant host without pathogenesis [1]. These symbiotic bacteria and fungi are called endophytes, and they benefit their host in two main aspects: promoting plant growth and protecting the host from invaders [2,3]. Our study focused on the second aspect in endophyte-assisted plant defense. It is commonly known that endophytic bacteria, like actinobacteria, produce a wide range of anti-infective and cytotoxic agents; one of the most notable examples is maytansine (Figure 1, left) [3,4]. It has shown potential antifungal properties [5] and is currently used as an anticancer drug to treat breast cancer [6,7]. However, our understanding of how endophytes organize maytansine production in plants remains uncertain [8], and the impacts of endophytic maytansine biosynthesis on secondary plant metabolites are likewise unclear. In this study, we explored these questions further.

Maytansine was first accepted as a plant metabolite after its discovery in 1972 from Ethiopian Celastraceae plants, namely *Gymnosporia serrata* (synonym *Maytenus serrata*) and *G. ovata* (synonym *M. ovata)* [9]. Interestingly, nearly thirty maytansine derivatives were later isolated from other plants; for example, *Putterlickia verrucosa* (Celastraceae), *Colubrina texensis* (Rhamnaceae) and *Trevia nudiflora* (Euphorbiaceae) [10]. In 1977, the first bacterial maytansine analog, ansamitocins, was reported from *Actinosynnema pretiosum* [11]. This discovery posed the question: was maytansine a plant or a microbial metabolite?

In 2003, Pullen et al. found an inconsistency in the producibility of maytansine in *Gymnosporia* and *Putterlikia* plant species [12]. Moreover, a clone from a maytansine-positive plant lost its ability to produce maytansine after vegetative propagation. None of the maytansine biosynthetic genes were detected from plant materials. The researchers suggested that the endophytes in plants responded to inconsistency in maytansine production [12]. Ten years later, Wing et al. confirmed Pullen’s suggestion by reporting the presence of the 16S rDNA of *A. pretiosum* in *P. verrucosa* plant materials [13]. A year later, Kusari et al. reported the existence of a maytansine biosynthetic gene (amino-hydroxybenzoic acid, AHBA, synthase) from endophytic bacteria isolated from *Putterlikia* roots. This evidence led to the conclusion that endophytic bacteria responded to maytansine production in the plants [14]. It is now accepted that endophytes produce maytansine; however, maytansine was absent in pure bacteria culture [10]. This information indicated a consortium—between either plant–endophyte, endophyte–endophyte, or both—that played an essential role in maytansine production in the plant.

In 2016, Kusari et al. presented groundbreaking information indicating that *G. serrata* (synonym. *M. serrata*), as a host plant, might produce AHBA as a precursor for the maytansine biosynthesis along with its endophytes [15]. Kusari et al. first reported an endophytic consortium organizing maytansine production inside the plant. In their report, a specific group of endophytes harboring in the bark was the only endophytic community that presented with the halogenase gene, responding to the halogenation step in maytansine biosynthesis (adding a chlorine atom to the maytansine structure). In contrast, other endophytes living in different plant organs carried only the AHBA synthase gene [15]. Unfortunately, they did not report how many endophytes were presented with the halogenase gene found in the bark’s endophytic community. Kusari et al. did not investigate the impact of maytansine biosynthesis on *Gymnosporia* plant secondary metabolite production. It is commonly known that all *Gymnosporia* plants produce a particular group of metabolites known as quinone-methide pentacyclic triterpenoids (QMTs) [16,17], and they link with plant defense, since they commonly show anti-infective properties against plant pathogens [18,19,20]. These questions remained unanswered.

**Figure 2:**
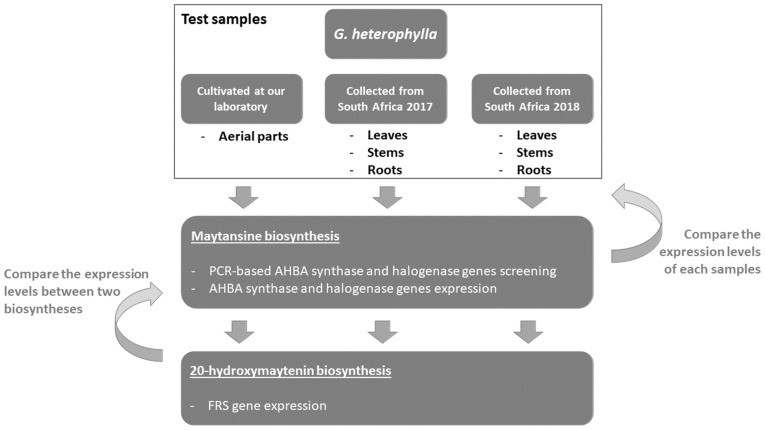
Experimental scheme showing the design and techniques applied in this study.

Our study aimed to address these questions. The experimental design and techniques applied in this study are presented in Figure 2. In detail, we first selected *G. heterophylla* as our model and used PCR-based genome mining as a tool to explore a diversity of maytansine-producible endophytic communities in *G. heterophylla*. We confirmed the producibility of maytansine from the detected endophytes using the gene expression approach. Second, we evaluated the impacts of maytansine biosynthesis on *Gymnosporia* plant secondary metabolite production, focusing on QMT metabolites produced by *G. heterophylla*. In addition, we found from our previous report that *G. heterophylla* could produce 20-hydroxymaytenin (Figure 1, right), a QMT [21]. Therefore, we selected the friedelin synthase (FRS) gene from 20-hydroxymaytenin biosynthesis as a plant metabolite gene and compared it with the endophytic maytansine biosynthetic genes. As a result, we found a specific group of endophytic bacteria responsible for producing AHBA, a precursor of maytansine. In addition, for the first time, we identified six clusters of the halogenase gene from endophytic communities, harboring specifically in the roots of *G. heterophylla*. In total, there were seven endophytes involved in maytansine production. Furthermore, our gene expression experiment suggested a stress trigger to activate maytansine biosynthesis from the endophytic communities. The same trigger upregulated the production of 20-hydroxymaytenin from *G. heterophylla*, a plant host.

In conclusion, our study presented new evidence confirming an endophytic consortium in maytansine production in *G. heterophylla*. We also reported a novel finding, showing a plant–endophyte interaction inducing upregulation of the 20-hydroxymaytenin biosynthetic (FRS) gene. This information will contribute to a better understanding of endophyte–endophyte and plant–endophyte interactions in response to bioactive metabolite production in plants.

## 2. Results

### 2.1. PCR-Based Genome Mining for Maytansine Biosynthetic Genes from G. heterophylla

#### 2.1.1. Amino-Hydroxybenzoic Acid (AHBA) Synthase Gene from *G. heterophylla*

**Figure 3:**
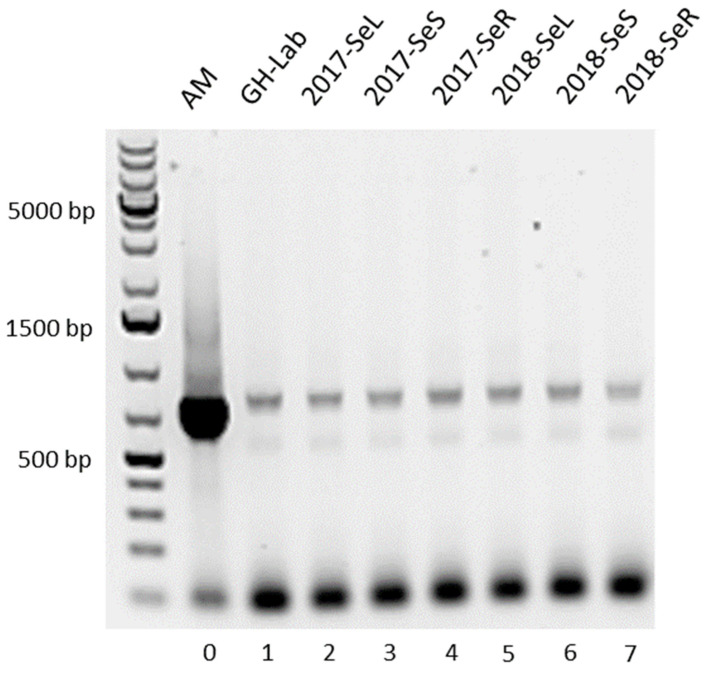
PCR products of the amplified AHBA synthase gene, spanning around 755 bp in different parts of 2017 and 2018 collections of *G. heterophylla*, originating from South Africa, compared to *A. mirum* (DSM 43827) as the reference. Our reference, *A. mirum* (AM), is indicated by 0, while 1 indicates the aerial part of *G. heterophylla* cultivated at our laboratory in Germany (GH-Lab). Furthermore, 2 to 4 indicate the leaves, stems, and roots of the 2017 collection of *G. heterophylla*, originating from South Africa (2017-SeL, 2017-SeS, and 2017-SeR), while 5 to 7 indicate the counterpart from the 2018 collection (2018-SeL, 2018-SeS, and 2018-SeR).

All *G. heterophylla* plant specimens were presented with a PCR product spanning around 755 bp, closing to our reference bacterium *A. mirum* (DSM 43827), as shown in Figure 3. However, there were two components deemed not ideal for our PCR results. The first was a high yield of the PCR product from the reference (sample 0). We used the same volume of the DNA template as in the other samples. However, our reference was a pure strain of *A. mirum,* while the other samples were a mixture of endophytes and *G. heterophylla* presenting with high diversity DNA templates. Therefore, the PCR reaction from the reference bacterium *A. mirum* provided a higher product yield than the other mixture samples when using the same amount of the DNA template.

The second was primer-dimers showing PCR products at the bottom of the gel and nonspecific binding shadow bands spanning around 650 bp. We used the AHBA synthase gene primers designed by Huita et al. [22]. This pair of primers was developed based on a conserved region of the amino acid sequences of the AHBA synthase enzyme from five different organisms to serve as primers for a PCR screening. Notably, *A. mirum* was not included in Huitu’s study. Therefore, based on our findings, these primers could be used, albeit not ideally, to amplify the AHBA synthase gene from *A. mirum*.

A nonspecific amplification found around 650 bp (a shadow band) could occur due to the complexity of the DNA templates, since samples number 1 to 7 consisted of a mixture of DNA templates between endophytes and *G. heterophylla*. Li and Yan [23] reported similar findings on a nonspecific binding from a mixture of DNA samples. Notably, these components can cause a false positive detection. Therefore, PCR products at approximately 750 bp were sequenced to verify our findings.

As a result, the obtained nucleotide sequences confirmed that the amplified PCR products were AHBA synthase genes. The AHBA synthase gene from *G. heterophylla* materials showed more than 97% homology to *A. mirum*. Finally, all obtained sequences were deposited on the NCBI database (NCBI accession numbers: OK649322 to OK649329). The AHBA synthase gene sequence alignment was provided in the supplementary file (Figure S1).

Later, we preliminary evaluated the functional producibility of the AHBA synthase from the obtained sequences. All sequences were translated to amino acids and aligned with known AHBA synthase proteins encoded by the rifK gene from *Amycolatopsis mediterranei* [23]. We found that some amino acids were altered. However, the proposed active amino acids involved in enzymatic functions were conserved. As a result, based on this finding, we initially expected a nonsignificant change in enzyme activity among the obtained sequences. However, more investigation (i.e., in vitro assay) is required to confirm our initial report. Finally, we provided the amino acids alignment of the AHBA synthase of each sequence in the supplementary file (Figure S2).

#### 2.1.2. Halogenase Gene from *G. heterophylla*

**Figure 4:**
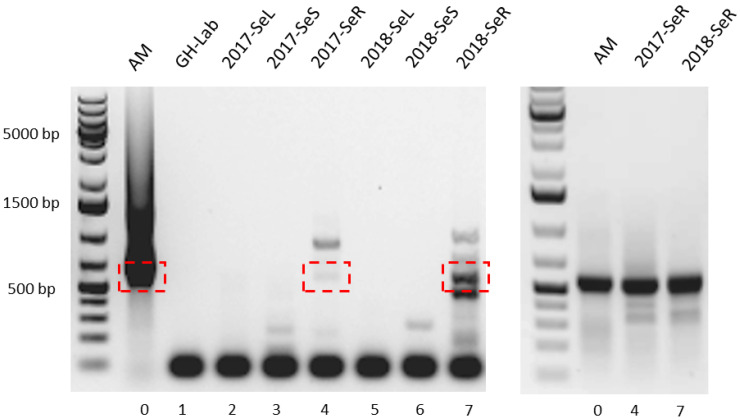
PCR products of the amplified halogenase genes, spanning around 550 bp in different parts of 2017 and 2018 collections of *G. heterophylla* originated from South Africa, compared to *A. mirum* (DSM 43827) as the reference. Of note: 0 indicates *A. mirum* (AM) while 1 indicates the aerial part of *G. heterophylla* cultivated at our laboratory in Germany (GH-Lab). Additionally, 2 to 4 indicate the leaves, stems, and roots parts of the 2017 collection of *G. heterophylla* originated from South Africa (2017-SeL, 2017-SeS, and 2017-SeR), while 5 to 7 indicate counterparts from the 2018 collection (2018-SeL, 2018-SeS, and 2018-SeR). The left-handed figure indicates the 1st amplification, while the right-handed one indicates the 2nd amplification, both from the isolated 1st PCR products (red boxes).

We obtained a PCR product spanning approximately 550 bp from the roots of *G. heterophylla* in both collections (2017 and 2018). However, the PCR product yields were too low, as presented in Figure 4, left. Therefore, we performed a second PCR with the same primers to improve the product. As a result, the second PCR reaction with the same set of primer improved the yields and the sensitivity of the PCR reaction significantly (Figure 4, right).

Notably, we observed a similar PCR result as in the earlier experiment from the first PCR reaction, as shown in Figure 4, left. A pure DNA template from *A. mirum* and a mixture of DNA templates from *G. heterophylla* and its endophytes were the cause of the observation. Therefore, the same explanation mentioned above can be applied here. Finally, the PCR products were cloned into the pGAM-T easy vector before sequencing to verify our findings.

**Figure 5:**
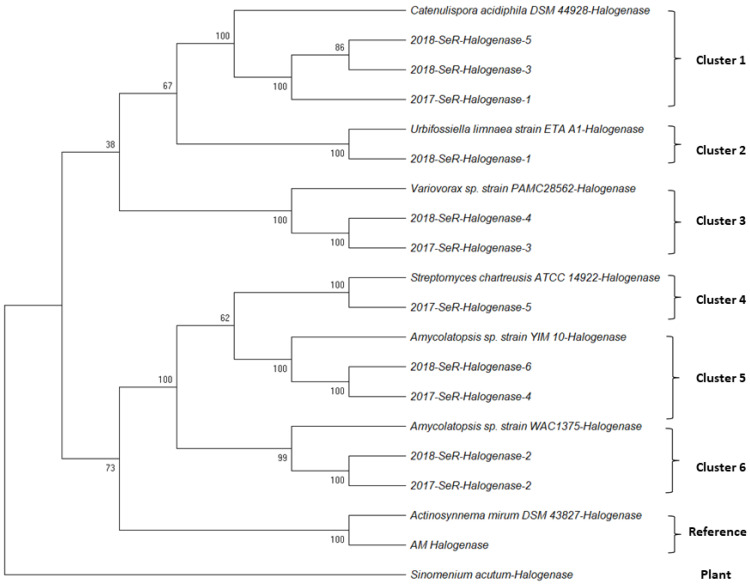
Phylogenetic tree of the obtained halogenase gene sequences from *G. heterophylla*, and homologous sequences from the NCBI database (https://www.ncbi.nlm.nih.gov/, accessed on 15 October 2021). This tree is deposited on the TreeBASE database (TreeBASE ID: Tr134221). Multiple alignments are performed using the Muscle tool, and the phylogenetic tree is generated using the UPGMA model from MEGA-X software (Version 10.0.4). The bootstrap values are shown on the branch based on 1000 pseudoreplicates. The distances of this evolutionary tree are calculated using the Maximum Composite Likelihood method. The halogenase gene from *Sinomenium acutum* is used as a plant gene.

As a result, eleven halogenase gene sequences were obtained from the 2017 and 2018 collections of *G. heterophylla*. Our nucleotide sequences showed an appreciable homology (78% to 86%) to the deposited halogenase genes on the NCBI database (https://www.ncbi.nlm.nih.gov/, accessed on 15 October 2021). We provided detail regarding the homologous sequences and the organisms in the supplementary file (Table S1). Later, all obtained sequences and the homologs were used to perform the phylogenetic analysis (Figure 5). We chose plant halogenase gene sequences from *Sinomenium acutum* [24] to exhibit no relationship between our obtained halogenase genes from the endophytic bacteria living inside *G. heterophylla* and the plant halogenase gene. Based on the authors’ best knowledge, we identified six different clusters of endophytic halogenase genes inside *G. heterophylla* for the first time. In addition, we found that specific halogenase genes only presented in a particular collection of *G. heterophylla* roots. For example, cluster 2 (*Urbifossiella limnaea* strain ETA A1) [25] occurred explicitly in the 2018 collection, whereas cluster 4 (*Streptomyces chartreusis* strain ATCC 14922) [26] only appeared in the 2017 collection. On the contrary, we noticed that some halogenase genes presented in both groups, such as cluster 1 (*Catenulispora acidiphila* strain DSM 44928), cluster 3 (*Variovorax* sp. strain PAMC28562) [27], cluster 5 (*Amycolatopsis* sp. strain YIM 10) [27], and cluster 6 (*Amycolatopsis* sp. strain WAC1375) [28]. The information here suggested a dynamic change of the endophytic communities in the root over the year. In this experiment, all obtained halogenase genes were deposited in the NCBI database (NCBI accession numbers: OK649330 to OK649336, OK754597 to OK754600, and OL944594), and the phylogenic tree was deposited on the TreeBASE database under TreeBASE ID: Tr134221 (http://purl.org/phylo/treebase/phylows/study/TB2:S28886, accessed on 9 October 2021). We provided all the details of the obtained halogenase gene sequences used to perform phylogenetic analysis in the supplementary file (Table S2).

### 2.2. Maytansine and 20-Hydroxymaytenin Biosynthetic Genes Expressions from Endophytic Communities and G. heterophylla

#### 2.2.1. AHBA Synthase and Halogenase Genes Expression from Endophytic Bacteria in *G. heterophylla*

**Figure 6:**
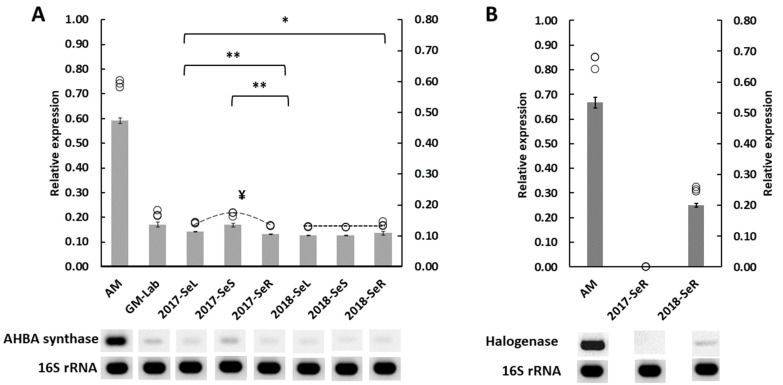
Maytansine’s biosynthetic genes expression. AHBA synthase gene expression is presented on the left (**A**), while halogenase gene expression is shown on the right (**B**). The relative gene expression is exhibited in the mean ± SD of triplicated samples (*n* = 3). AM indicates *A. mirum* (DSM 43827) as a reference. GH-Lab indicated *G. heterophylla* cultivated in our laboratory, Germany. 2017-SeL, 2017-SeS, and 2017-SeR indicate the leaves, stems, and roots parts of the 2017 collection of *G. heterophylla* originated from South Africa, while 2018-SeL, 2018-SeS, and 2018-SeR indicate the counterpart from the 2018 collection. Additionally, 16S rRNA is used as a housekeeping gene. The bar graph uses the *x*-axis on the right side while O indicates each sample data point, using the *x*-axis of the left side. * indicates the statistic significant by student *t*-test (one-tail) with *p*-value ≤ 0.05. ** indicates the statistic significant by one-way ANOVA with *p*-value ≤ 0.05. ^¥^ indicates the highest AHBA synthase gene expression.

We evaluated the maytansine producibility in the endophytic communities living in *G. heterophylla*, discovered in an earlier experiment using gene expression. We found that both AHBA synthase and halogenase genes were expressed, as shown in Figure 6. However, only bacterial endophytes inside *G. heterophylla* collected in 2018 could produce maytansine, based on our experiment here. While the 2017 *G. heterophylla* could not synthesize maytansine, we observed a significantly higher AHBA synthase gene expression level from the 2017 plant than its 2018 counterpart (Figure 6A). Moreover, the highest AHBA synthase expression level was exhibited in the stems of the 2017 plant, even though the halogenase gene was not expressed from the roots of the 2017 collection, as presented in Figure 6B. Notably, there was an insignificant level of the AHBA synthase gene expressed among leaves, stems, and roots from the endophytic AHBA synthase gene in *G. heterophylla* materials collected in 2018. Furthermore, we observed a slightly higher expression level of the halogenase gene than the AHBA synthase gene in our reference bacterium, *A. mirum*. A similar trend was found in *G. heterophylla* collected in 2018 but with a higher magnitude; approximately double expression level of the halogenase gene versus the AHBA synthase gene from bacterial endophytes. To our knowledge, this was the first report to present maytansine biosynthetic genes expressed from the endophytic communities harboring in plant materials [12,13,14,15,27]. We provided statistical detail regarding this experiment in a supplementary file (Table S3).

#### 2.2.2. Friedelin Synthase or FRS Gene Expression from *G. heterophylla*

**Figure 7:**
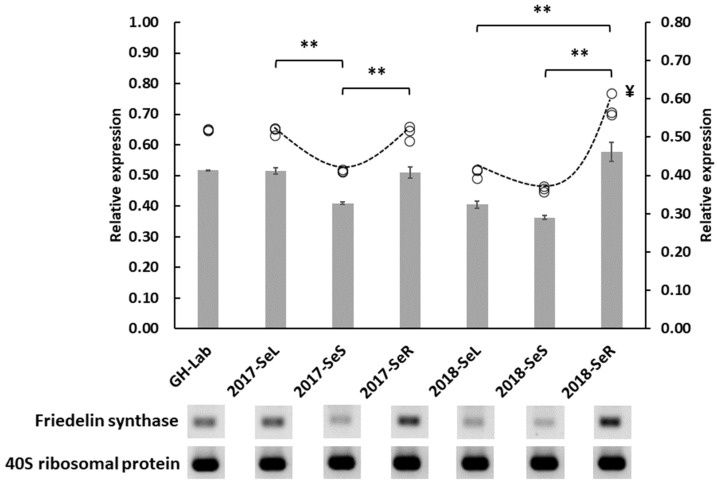
FRS gene expression of *G. heterophylla* from our laboratory and the 2017 and 2018 collections originating from South Africa. GH-Lab indicates *G. heterophylla* cultivated in our laboratory, Germany; 2017-SeL, 2017-SeS, and 2017-SeR indicate the leaves, stems, and roots of the 2017 collection of *G. heterophylla* originating from South Africa; 2018-SeL, 2018-SeS, and 2018-SeR indicate the counterparts from the 2018 collection. Additionally, 40S ribosomal protein is used as a housekeeping gene. The bar graph uses the *x*-axis on the right side while O indicates each sample data point, using the *x*-axis of the left side. ** indicates the statistic significantly by one-way ANOVA with *p*-value ≤ 0.05. ^¥^ indicates the highest FRS gene expression level.

The expression levels of the FRS genes of all plant materials were observed to evaluate their relationship to the maytansine biosynthetic gene from the endophytes that we found in our previous experiment. All obtained data were visualized in Figure 7. Interestingly, we found that the roots from the 2018 collection expressed the highest levels of the FRS gene across all samples. These roots are the same ones determined to be capable of producing maytansine; both AHBA synthase and halogenase genes were expressed (Figure 6). Notably, we also observed a similar expression pattern (V-shape-like pattern) from both collections of *G. heterophylla*, showing that the stems of both groups expressed the lowest levels of the FRS gene while the other plant organs expressed more. Although there was no statistically significant difference between the FRS gene expressions of the collections, the 2018 roots expressed the FRS gene more excessively than their 2017 counterparts, leading to a nonsymmetric V-shape with one end high. In addition, compared within the same group, the FRS gene level from the roots was significantly higher than the levels from leaves and stems. Finally, the 2018 roots were the only sample that expressed higher levels of the FRS gene than the *G. heterophylla* cultivated in our laboratory. The other samples expressed at equal or lower levels. To the extent of our knowledge, we were the first to compare the expression of the FRS gene from the plant organs (i.e., leaves, stems, and roots) of *G. heterophylla* and link it with endophytic maytansine biosynthetic genes. All statistical details are provided in the supplementary file (Table S4).

### 2.3. Transmission of Maytansine Producible Endophytes to a Vegetatively Cloned G. heterophylla Plant

**Figure 8:**
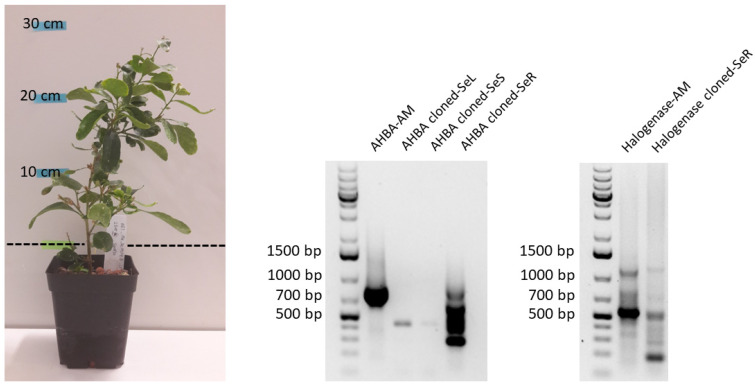
A six-month-old vegetatively cloned *G. heterophylla* plant from a stem of *G. heterophylla* collected in 2018, originating from South Africa (**left**). PCR products of the amplified AHBA synthase genes spanning around 755 bp in the different parts of the cloned *G. heterophylla* (**middle**). PCR products of the amplified halogenase genes spanning around 550 bp in the different parts of the cloned *G. heterophylla* (**right**). AM indicates *A. mirum* (DSM 43827) as the reference. Cloned-SeL, cloned-SeS, and cloned-SeR indicate leaves, stems, and roots of the vegetatively cloned *G. heterophylla*.

A cloned plant was generated from the stem of the 2018 *G. heterophylla* plant via vegetative propagation using the cutting technique. This cloned plant was cultivated in our laboratory in Germany for six months, as shown in Figure 8. Later, we performed the PCR-based genome mining experiment from the leaves, stems, and roots of the cloned *G. heterophylla* to evaluate the transmissibility of the maytansine-producible endophytes from the 2018 mother plant. Interestingly, for the first time, we found that both endophytic bacteria carrying AHBA synthase and halogenase genes were presented in the vegetatively cloned *G. heterophylla* (Figure 8, middle and right). This finding indicated that maytansine-producible endophytic communities could be passed from the mother to the cloned plant through vegetative propagation. However, once again, our PCR electrophoresis gel indicated a nonideal PCR reaction. However, we provided a possible explanation for this in the previous section.

## 3. Discussion

Maytansine and its derivatives are now known as bacterial metabolites and can be found in actinobacteria like *Actionosynnema* spp. It is commonly known that *Actionosynnema* spp. is an endophytic bacteria living inside plants. However, pure cultivation of *Actionosynnema* spp. cannot produce maytansine precisely but only synthesize its analog, ansomitocin P3. Therefore, plant–endophyte interaction plays a crucial role in maytansine production. However, our understanding regarding this issue is incomplete.

Our results indicated that a specific group of endophytic bacteria carried the AHBA synthase gene and lived inside multiple organs of *G. heterophylla*, e.g., leaves, stems, and roots. We were able to amplify a PCR product in all samples, and all obtained endophytic AHBA synthase genes showed 97–99% homology to the AHBA synthase gene from *A. mirum* (our bacteria reference). Furthermore, based on our preliminary translated amino acids evaluation, differences in nucleotide sequences might not impact enzyme functionality. We were not the first to report the presence of the AHBA synthase-positive gene in multiple plant organs in the Celastraceae plant. In 2016, Kusari et al. amplified the AHBA synthase gene from all *G. serrata* organs [15]. They also found that maytansine was present in the samples using HPLC-MS analysis. In their study, each plant organ presented with different AHBA synthase genes. They proposed that the *G. serrata* plant might also carry the AHBA synthase gene and produce the maytansine precursor with endophytes [15]. However, this was not the case in our study; all of our obtained sequences showed high homology (≥97%) to *A. mirum*.

On the other hand, we were the first to report six different halogenase gene clusters from endophytic communities harboring inside *G. heterophylla* roots. It is noteworthy that most of the endophytes we found could biosynthesize maytansine. Based on the KEGG (Kyoto Encyclopedia of Genes and Genomes) database (https://www.genome.jp/kegg/, accessed on 15 October 2021), three endophytes—*Catenulispora acidiphila* strain DSM 44928, *Streptomyces chartreusis* strain ATCC 14922, and *Amycolatopsis* sp. strain YIM 10—from Clusters 1, 4, and 5 presented with the maytansinoids biosynthetic genes, while the other two endophytes—*Urbifossiella limnaea* strain ETA A1 and *Variovorax* sp. strain PAMC28562—from Clusters 2 and 3 did not. *Amycolatopsis* sp. strain WAC1375 from cluster 6 was not listed in the KEGG database [28,29]. It was deemed likely that *Amycolatopsis* sp. strain WAC1375 also carried the maytansinoids biosynthetic genes, like *Amycolatopsis* sp. strain YIM 10 from Cluster 5, a close species, after careful evaluation of the deposited genome data of *Amycolatopsis* sp. strain WAC1375 on the NCBI database (https://www.ncbi.nlm.nih.gov/, accessed on 15 October 2021) [30]. Interestingly, we observed some endophytic bacteria appeared in the roots in the 2017 collection and later disappeared in the 2018 collection. However, we also detected some endophytic communities which were present in both samples. Our findings indicated a specific and dynamic change in a group of roots endophytes over time.

We evaluated both AHBA synthase and halogenase gene expression levels in *G. heterophylla* samples. These data represent the first reports of endophytic maytansine biosynthetic genes’ expression from Celastraceae plant materials. We found that the AHBA synthase was expressed from the endophytic bacteria in all samples, and the halogenase gene was particularly expressed from the endophytic communities in the roots. Only *G. heterophylla* collected in 2018 expressed both genes—showing that it could produce maytansine.

We found two distinct points of interest within the study. First, we observed a higher expression level from the halogenase gene than the AHBA synthase gene in endophytic communities living inside the 2018 *G. heterophylla*. A similar finding was also noted in *A. mirum*, our reference strain. The high expression level of the halogenase gene might link with its physiological function as a biosynthetic activating switch. Only a few studies have investigated the expression pattern of maytansine biosynthetic genes, and none of these studies evaluated the impact of halogenase on maytansine production [29,30]. Our knowledge regarding this effect is limited and needs more investigation to confirm our proposed function as a biological activating switch of the halogenase gene.

In the nonproducible maytansine *G. heterophylla*, the AHBA synthase gene was expressed more from an endophyte than in the producible one. Our result thus opposed previous reports showing that the upregulation of the AHBA synthase gene increased the production of the maytansine derivative ansamitocin P3, [30,31]. In Li’s study, the asm18 gene, encoding a regulatory protein, was overexpressed in *A. pretiosum*, resulting in a selective upregulation of the AHBA synthase gene and leading to enhanced ansamitocin P3 production [31]. Moreover, Gao’s study used a combination of glucose and glycerol as a carbon source, mixing in a cultivation medium of *A. pretiosum* culture to increase the production of ansamitocin P3 by elevating AHBA synthase gene expression. Both Li’s and Gao’s results were from a straightforward system and single strain cultivation. Our results came from endophytic communities harbored inside a plant, a much more complex system. Therefore, circumstantial differences between previous studies and our study might explain the altered AHBA synthase gene expression. In addition, the higher expression levels of the AHBA synthase when the halogenase gene did not express might suggest another ecological function of AHBA in a plant, i.e., more than just as a precursor for maytansine production.

**Figure 9:**
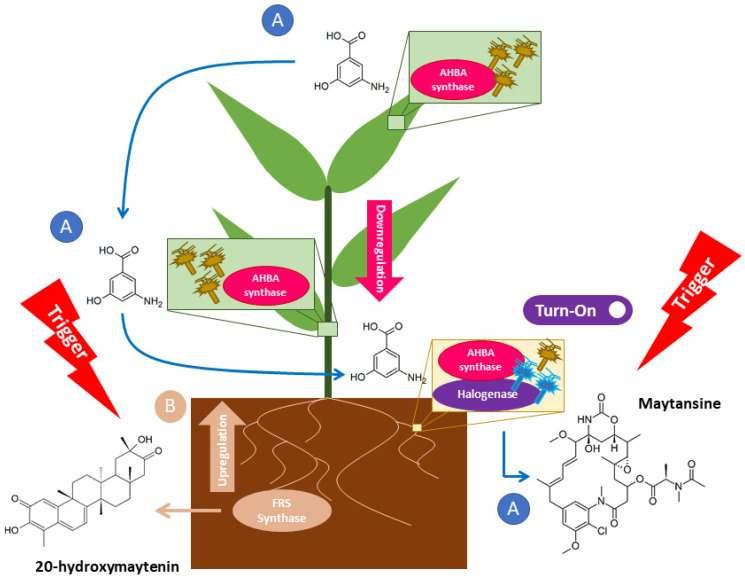
Proposed mechanistic response and relationship between the maytansine biosynthesis from endophytic communities and the 20-hydroxymaytenin biosynthesis from *G. heterophylla*, a plant host. A indicates the downregulation of the AHBA synthase gene and the activation of the halogenase from endophytes in different organs of *G. heterophylla*, and B indicates the upregulation of the FRS gene from the 20-hydroxymaytenin biosynthesis in the *G. heterophylla* roots. Blue arrows indicate the flow of AHBA transportation from the leaves to roots.

We hypothesized that a particular stress trigger might activate halogenase gene expression to prompt maytansine biosynthesis from endophytes in *G. heterophylla*. To test our hypothesis, we evaluated the production of 20-hydroxymaytenin using the same gene expression experiment, since we found, in our previous work, that 20-hydroxymaytenin was linked with antifungal activity and related to plant defense [21]. We selected the FRS gene from the 20-hydroxymaytenin biosynthesis as a marker. Later, we found that the expression of the FRS gene was upregulated in the roots of the 2018 *G. heterophylla*, the same sample capable of producing maytansine by endophytic communities. Based on the best authors’ knowledge, we were the first to observe and report this relationship between the endophytic maytansine and the plant 20-hydroxymaytenin biosyntheses. Previously, Pavarini and colleagues showed that both biotic and abiotic stimuli affected the production of maytenin (a parent structure of 20-hydroxymaytenin) in the *Gymnosperia* plant. However, biotic stimuli demonstrated better impact than abiotic stimuli [31]. This was supported by the latest study from Inácio in 2019, reporting that a co-cultivation between *G. ilicifolia* and *Bacillus megaterium* increased maytenin production by 24 times over the control without *B. megaterium* [32]. Combining all data from recent works in the literature [31,32], our previous study [21], and the current study, we proposed a possible mechanistic response of endophytic communities and *G. heterophylla* to the stress triggers activating maytansine production and upregulating 20-hydroxymaytenin biosynthesis simultaneously (Figure 9). In Eckelmann’s study, the researchers proposed something similar, stating that it could be an endophytic-associated trigger activating maytansine biosynthesis during and after the propagation processes to protect the seeds and young plants—thus providing a greater chance of survival against natural pathogens or herbivores [27].

Our data provided a more integrative response, leading to a better understanding of endophyte–endophyte and plant–endophyte interactions related to maytansine and 20-hydroxymaytenin biosyntheses. It is worth noting that we did not proceed with chemical analysis to determine the concentration of maytansine and 20-hydroxymaytenin in *G. heterophylla* plants originating from South Africa in both 2017 and 2018 collections due to the minimal weight of samples.

We evaluated the transmissibility of the maytansine-producible endophytes in *G. heterophylla*. We used the young branch of a stem from the 2018 *G. heterophylla* presented with the AHBA synthase gene-positive for a vegetative propagation by a cutting technique. After six months of cultivation (Figure 8), the vegetatively cloned *G. heterophylla* was terminated, and the presence of the AHBA synthase gene was evaluated. AHBA synthase was detected in the roots of the cloned plant, but not in the stem as in the original plant. This indicated that endophytic bacteria carrying the AHBA synthase gene moved from the branch to the roots after regeneration. Pereira et al. reported that roots cultivated in a greenhouse with high sugars and amino acid contents were more attractive to endophytes because they need a carbon source and amino acids to grow [33]. Vranova and her colleagues reported that young roots produced more sugars and amino acid concentrations than old ones [34]. This could explain the relocation of the AHBA synthase gene-positive endophytes to the newly generated cloned’s roots. We did not find that the AHBA synthase gene was expressed in the cloned *G. heterophylla*. It seems that the cloned plant was cultivated in a safer environment (i.e., the laboratory) than the natural habitat of the original plant (South Africa). The AHBA synthase gene was expressed from *G. heterophylla* cultivated in our laboratory (GM-Lab) in the same conditions as the cloned plant, as depicted in Figure 5. It is worth noting that the cloned plant was cultivated using the hydroponic method, i.e., non-soil cultivation, while *G. heterophylla* from our laboratory (GM-Lab) was grown in a regular potting soil mix. Since hydroponic cultivation faces less abiotic and biotic stresses than soil-based cultivation [35,36], the difference between these methods may impact the expression of the AHBA synthase gene between the cloned and *G. heterophylla* from our laboratory (GM-Lab).

The halogenase gene was detected from the roots of the cloned plant when the same halogenase gene was absent in the mother plant. There are two possible explanations for this The first is the cloned plants uptaking the halogenase gene-positive bacteria from the surrounding environment, e.g., soil, a rich source of bacteria [37]. We reject this hypothesis because our cloned plant was cultivated using clay pebbles (synthetic non-soil materials) under laboratory conditions. Second, there could have been a change in the endophytic population inside the cloned plant roots. A recent report from Saleh’s study supported this explanation, reporting that the roots of *Brachypodium distachyon* produced and secreted particular organic acids favoring a specific endophytic bacteria strain over the others [38]. By quorum sensing, like autoinducer-2, the endophyte could be positively stimulated to colonize in plant tissue like roots, as reported by Xiong et al. [39]. Another recent report from Forte et al. showed that a pattern of endophytes was changed over the propagation from one generation to another generation. Forte suggested that endophytes adapted to the plant’s metabolites and defense mechanisms over generation [40]. We found that the occurrence of the halogenase gene in the cloned *G. heterophylla* plant came from the alteration of the endophytic population, as reported by Saleh, Xiong, and Forte [38,40]. Our study thus presented a new piece of evidence showing that maytansine-producible endophytes can transmit from a mother plant to a cloned plant via vegetative propagation.

## 4. Materials and Methods

### 4.1. Plant Materials

We used three different *G. heterophylla* plants specimens in this study, including *G. heterophylla* cultivated at our laboratory, Technical Biochemistry Laboratory, Faculty of Biochemical and Chemical Engineering, Technical University Dortmund University, Dortmund, Germany (voucher number: GH-CHEM-2017), the authentic materials from our previous study [21], and two collections of *G. heterophylla* from Seweweekspoort, South Africa, dating from 2017 (voucher number: GH-Se-2017) and 2018 (voucher number: GH-Se-2018). Both *G. heterophylla* collected in 2017 and 2018 were sampled independently and identified by Mr. Ulrich Feiter, Parceval, Wellington, South Africa.

After receiving the plant samples, two DNA barcodes, i.e., rcbL and matK genes, were amplified, sequenced, and compared to the authentic *G. heterophylla* used in our previous report to confirm the plant species [21]. The obtained sequences were deposited in the NCBI database (https://www.ncbi.nlm.nih.gov/, accessed on 9 October 2021). Later, the multiple nucleotide sequences were presented in the Supplementary Materials (Figures S3 and S4). The obtained sequences were 100% identical to the authentic *G. heterophylla*, and thus, the collected plant materials from Seweweekspoort, South Africa, in 2017 and 2018 were confirmed as *G. heterophylla*.

### 4.2. PCR-Based Genome Mining Experiment Amplifying Maytansine Biosynthetic Genes from G. heterophylla Originating from South Africa

We performed an experiment by following Kusari et al. [13,14]. The following PCR-based genome mining experiment was described briefly and divided into three steps, as presented below.

#### 4.2.1. Preparation of *G. heterophylla* Plant Materials

The obtained plant samples were washed with running tap water to remove attaching dirt. After drying at room temperature for 20 min, the samples were cut into small pieces, approximately 10 mm in length. Surface sterilization was applied to remove microorganisms from the samples by submerging them in 70% ethanol for 1 min, followed by 1.3 M sodium hypochlorite (3 to 5% active chlorine) for 3 min, and additional immersion in 70% ethanol for 1 min. Both 70% ethanol and sodium hypochlorite were used as biocidal agents. Sterile ultrapure water was used to rinse the excessive agents. Finally, the samples were dried at room temperature for 20 min [14,15].

#### 4.2.2. Plant Genomic DNA Extraction and PCR Amplification for AHBA Synthase Gene from *G. heterophylla*

The surface-sterilized *G. heterophylla* samples from earlier were used here. First, the samples were homogenized using liquid nitrogen before the total genomic DNA was extracted. The total plant genomic DNA from the different plant organs—i.e., leaves, stems, and roots, of *G. heterophylla*—was extracted by Macherey Nagel Nucleospin Plant II (Düren, Germany), using spin silica-based column DNA extraction. The plant genomic DNA extraction was performed strictly following the manufacturer’s guidelines.

We used a primer pair to amplify the AHBA synthase gene designed for a PCR-based screening based on the bacterial protein’s conserved amino acids region from Huitu’s study [22]. In previous studies from Kusari et al., these primers were able to successfully amplify the AHBA synthase gene from plant materials [14,15]. Therefore, we decided to follow Huit et al. and Kusari et al. [14,15,22]. The used primer pair was defined as a forward primer (AHBA-F: AGAGGATCCTTCGAGCRSGAGTTCGC) and a reverse primer (AHBA-R: GCAGGATCCGGAMCATSGCCATGTAG) [14,15,22]. The Red Taq polymerase Master Mix (1.1x) from VWR Life Science (Denmark) was used to amplify the desired PCR product. We prepared a total of 50 µL of a PCR reaction mixture, strictly following the manufacturer’s guidelines. The PCR reaction solution used consisted of 0.5 µL of 100 µM of each primer, 2 µL of the plant’s total genomic DNA (50 to 160 ng/µL), 2 µL of the sterilized water, and 45 µL of the polymerase master mix. The concentration of total genomic DNA was in the recommended range of the Red Taq polymerase from the company (in a range of 50 to 500 ng in total). The PCR cycling consisted of an initial denaturation at 95 °C for 120 s, 35 cycles of denaturation at 95 °C for 30 s, annealing at 69 °C for 40 s, and elongation at 72 °C for 60 s. Lastly, the final elongation was done at 72 °C for 5 min. For reference, *A. mirum* strain DSM-43827 was used. The amplified PCR product, spanning around 755 bp, was checked by agarose electrophoresis. In the end, the PCR product was purified using Macherey Nagel Gel and PCR Clean-up (Düren, Germany) and sequenced by Microsynth/Seqlab (Goettingen, Germany). Finally, the obtained sequences were blasted against the NCBI database (https://blast.ncbi.nlm.nih.gov/Blast.cgi, accessed on 1 October 2021).

#### 4.2.3. PCR Amplification for Halogenase Gene from *G. heterophylla* and the Vector-Cloned Library Construction

For halogenase gene amplification, the used primer pair was defined as the halogenase-forward primer (Halo-F: TTCCCSCGSTACCASATCGGSGAG) and the halogenase-reverse primer (Halo-R: GSGGGATSWMCCAGWACCASCC) [15,41]. The same PCR protocol as earlier was also applied here, and only the annealing temperature was reduced to the optimum temperature of 59 °C. The PCR product spanning around 550 bp was expected [15]. The obtained PCR product was purified using the same extraction kit as the previous experiment and cloned into the pGEM-T easy vector (Promega, Madison, WI, USA), strictly following the manufacturer’s instructions. The JM109 *Escherichia coli* competent cells that came with the vector were transformed with the ligation-cloned product earlier and placed on the lysogeny broth (LB) agar supplemented with ampicillin/IPGT/X-gal. Ampicillin-resistance *E. coli* colonies with white color were picked randomly and we performed a colony-PCR to check the correct insert length. The colony that provided the correct size was cultivated overnight at 37 °C on LB liquid medium supplemented with ampicillin [42]. Later, the plasmid was isolated from the cultivated *E. coli* using Macherey Nagel NucleoSpin plasmid, mini kit (Düren, Germany), and sequenced by Microsynth/Seqlab (Goettingen, Germany). All obtained sequences were blasted against the NCBI database (https://blast.ncbi.nlm.nih.gov/Blast.cgi, accessed on 1 October 2021).

### 4.3. Maytasine and 20-Hydroxymaytenin Biosynthetic Genes Expression

#### 4.3.1. Maytansine Biosynthetic (AHBA Synthase and Halogenase) Genes Expression

The total plant RNA kit from Macherey Nagel (Düren, Germany) was used to extract the total RNA materials from the leaves, stems, and roots of the surface-sterilized *G. heterophylla* samples following the manufacturer’s instruction strictly. The obtained RNA was converted to the cDNA using Luna Universal qPCR Master Mix (New England Biolabs, Frankfurt, Germany) before performing the PCR reaction. The same primers and annealing temperature used in the previous experiments were applied here. The 16S ribosomal RNA gene was used as the housekeeping gene [43,44]. The relative gene expression was calculated using the intensity of the gene expression of the AHBA synthase and halogenase genes as a target gene against the housekeeping gene expression shown in Equation (1). ImageJ software version 1.52a was used to calculate the intensity [45]. Each sample was tested in triplicates (*n* = 3), and each relative gene expression value was presented in the standard form of mean ± SD.

(1) $$\mathrm{Relative}\;\mathrm{gene}\;\mathrm{expression}=\frac{\mathrm{Intensity}\;\mathrm{of}\;\mathrm{the}\;\mathrm{target}\;\mathrm{gene}\;\mathrm{expression}}{\mathrm{Intensity}\;\mathrm{of}\;\mathrm{the}\;\mathrm{housekeeping}\;\mathrm{gene}\;\mathrm{expression}}$$

#### 4.3.2. 20-Hydroxymaytenin Biosynthetic (FRS) Gene Expression

The same cDNA samples as above were also applied here. In addition, we used a primer pair from our previous report to amplify the FRS gene [21]. The FRS gene primers were presented as FRS-forward primer (FRS-F: ATGACTTTGTTGGCAGGCAG) and FRS-reverse primer (FRS-R: TGCGATGTTCCGGAGTGATA). The annealing temperature of these primers was set at 70 °C. The 40S ribosomal protein gene was used as a housekeeping gene [21]. A similar procedure, mentioned above, was applied here to calculate the relative gene expression with the same sample size (*n* = 3).

#### 4.3.3. Bioinformatic and Statistical Analysis

We used the Clone Manager version 9 Professional Edition to perform multiple sequences alignment and the Mega-X program version 10.0.4 to perform the phylogenetic analysis [46]. In addition, we used the UPGMA method to generate the tree and 1000 pseudoreplicates as the bootstrap value. Finally, we selected the Maximum Composite Likelihood method to distance the branch of the tree. All sequence datasets used here are provided in the supplementary file (Tables S1 and S2). For statistics, we used the data analysis package from Microsoft Excel to calculate means and SD, and Student’ *t*-test to determine statistical significance at the *p*-value of 0.05 (one-tail) to compare between year collection samples [47]. In addition, we used the Jmovi program (version 1.2.27) for the ANOVA analysis to compare subgroups, i.e., leaves, stems, and roots, of both collections. Details regarding the statistical analyses are presented in the supplementary file (Tables S3 and S4).

### 4.4. Data Availability

Every nucleotide sequence obtained in this study was deposited into the NCBI database (https://www.ncbi.nlm.nih.gov/, accessed on 15 October 2021). The NCBI accession numbers of the obtained AHBA synthase gene were OK649322 to OK649329. In addition, starting from OK649330 to OK649336, OK754597 to OK754600, and OL944594 were the NCBI accession numbers of the acquired halogenase gene. Finally, the NCBI accession numbers of the DNA barcoding of *G. heterophylla* started from OK649337 to OK649350. Furthermore, the constructed phylogenic tree was also deposited on the TreeBASE database under the TreeBASE ID: TB2:S28886. (http://purl.org/phylo/treebase/phylows/study/TB2:S28886, accessed on 9 October 2021).

## 5. Conclusions

This study demonstrated that the *G. heterophylla* plant did not support maytansine biosynthesis after providing AHBA as a starter. We found that the AHBA synthase gene presented in the *G. heterophylla* plant materials was from endophytic bacteria living inside the plant, not from the plant itself. We also identified six different clusters of the halogenase gene from endophytic bacterial communities in the roots of *G. heterophylla* collected from South Africa in 2017 and 2018. Our data showed a unique halogenase gene cluster presenting in only a specific collection (either the 2017 or 2018 collection), indicating a dynamic change of endophytic bacterial communities over the year. Notably, only *G. heterophylla* collected from South Africa in 2018 expressed both the AHBA synthase and halogenase genes, showing that maytansine could be produced. This information suggested that it might have been a specific trigger present in 2018 that activated maytansine biosynthesis which did not present in the 2017 collection. It is worth noting that 20-hydroxymaytenin biosynthesis (FRS gene) was also upregulated in *G. heterophylla* from the 2018 collection. This finding implied that the similar trigger activating maytansine biosynthesis also upregulated the plant’s FRS gene expression. To the authors’ best knowledge, this study marked the first time six different halogenase genes was reported from *G. heterophylla,* and that the correlation between maytansine and 20-hydroxymaytenin biosyntheses from endophytic communities and *G. heterophylla*, a plant host, was observed.

## Data Availability

Data reported are available in the supplementary material.

## Supplementary Materials

The following supporting information can be downloaded at: https://www.mdpi.com/article/10.3390/plants11030321/s1, Figure S1: Multiple alignments of amplified AHBA synthase gene obtained from leaves stems, and roots of *G. heterophylla* originated from South Africa from 2017 and 2018 collections, Figure S2: Multiple amino acid alignments of the translated AHBA synthase gene obtained from leaves stems, and roots of G. heterophylla originated from South Africa and the reference AHBA synthase enzyme encoded by the rifK gene from *Amycolatopsis mediterranei*, Figure S3: Multiple alignments of amplified rcbL gene obtained from leaves stems, and roots of *G. heterophylla* originated from South Africa from 2017 and 2018 collections compared to the authentic *G. heterophylla* cultivated in our laboratory in Germany, Figure S4: Multiple alignments of amplified matK gene obtained from leaves stems, and roots of *G. heterophylla* originated from South Africa from 2017 and 2018 collections compared to the authentic *G. heterophylla* cultivated in our laboratory in Germany, Table S1: Overview of the halogenase gene received from *A. mirum* (our reference) and presented in the roots of *G. heterophylla* collected from South Africa, Table S2: Obtained halogenase gene sequences from *A. mirum* (our reference) and the roots of *G. heterophylla* collected from South Africa, Table S3: Statistic analysis of the AHBA synthase gene expression of *G. heterophylla* collected from South Africa in 2017 and 2018 and cultivated at our laboratory in Germany, Table S4: Statistic analysis of the FRS gene expression of *G. heterophylla* collected from South Africa in 2017 and 2018 and cultivated at our laboratory in Germany.
