# Supplementary material for: Genome Mining and Gene Expression Reveal Maytansine Biosynthetic Genes from Endophytic Communities Living inside Gymnosporia heterophylla (Eckl. and Zeyh.) Loes. and the Relationship with the Plant Biosynthetic Gene, Friedelin Synthase
Source: Plants (Basel). 2022 Jan 25;11(3):321. doi: 10.3390/plants11030321 (PMC8840412; doi:10.3390/plants11030321)
Supplement: Supplementary file 1 [file plants-11-00321-s001.zip › plants-1501681-supplementary.pdf]

Supplementary file

Table of contents

Figure S1. Multiple alignments of amplified AHBA synthase gene obtained from leaves stems, and roots of *G. heterophylla* originated from South Africa from 2017 and 2018 collections.

Figure S2. Multiple amino acid alignments of the translated AHBA synthase gene obtained from leaves stems, and roots of *G. heterophylla* originated from South Africa and the reference AHBA synthase enzyme encoded by the rifK gene from *Amycolatopsis mediterranei*.

Figure S3. Multiple alignments of amplified rcbL gene obtained from leaves stems, and roots of *G. heterophylla* originated from South Africa from 2017 and 2018 collections compared to the authentic *G. heterophylla* cultivated in our laboratory, Germany.

Figure S4. Multiple alignments of amplified matK gene obtained from leaves stems, and roots of *G. heterophylla* originated from South Africa from 2017 and 2018 collections compared to the authentic *G. heterophylla* cultivated in our laboratory, Germany.

Table S1. Overview of the halogenase gene received from *A. mirum* (our reference) and presented in the roots of *G. heterophylla* collected from South Africa.

Table S2. Obtained halogenase gene sequences from *A. mirum* (our reference), and the roots of *G. heterophylla* collected from South Africa,

Table S3. Statistic analysis of the AHBA synthase gene expression of *G. heterophylla* collected from South Africa in 2017 and 2018 and cultivated at our laboratory in Germany,

Table S4. Statistic analysis of the FRS gene expression of *G. heterophylla* collected from South Africa in 2017 and 2018 and cultivated at our laboratory in Germany.

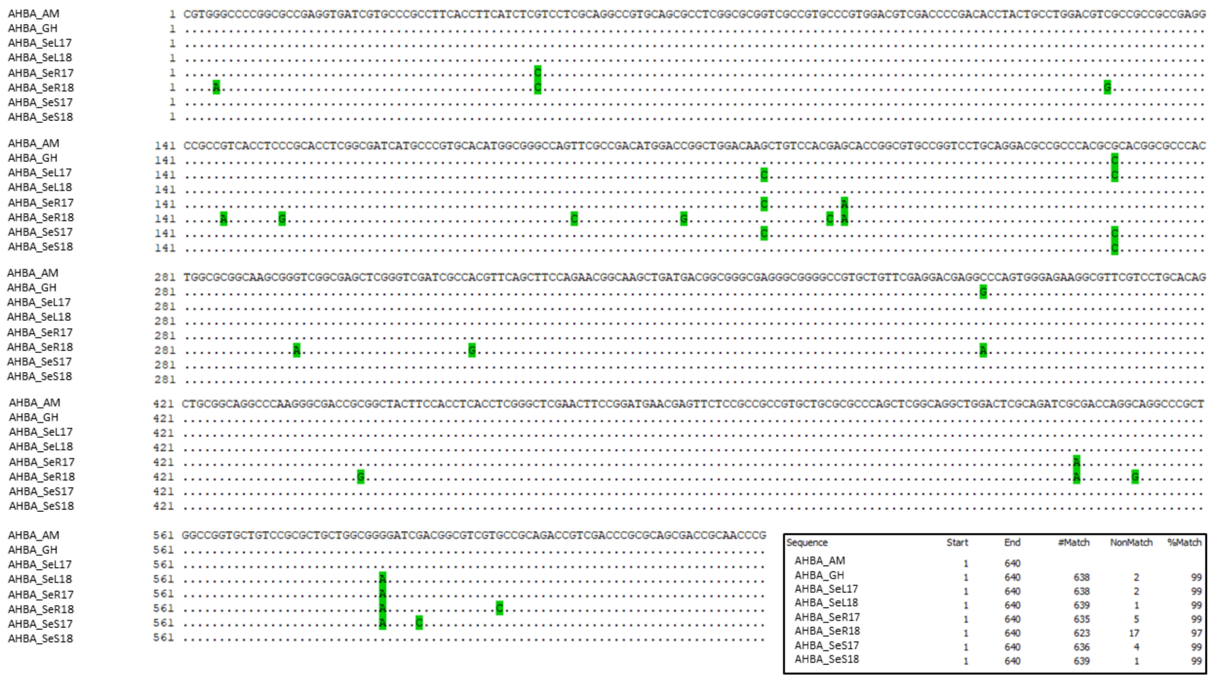

Figure S1. A multiple alignments of amplified AHBA synthase gene obtained from leaves stems, and roots of *G. heterophylla* originated from South Africa from 2017 and 2018 collections using global alignment from Clone Manager 9 Professional edition. The green highlighted indicates non-matching nucleotides, while the dot indicates the identical nucleotide. Am = *Actinosynnema mirum* (DMS 43827), the microbial reference. QH = *G. heterophylla* cultivating in our laboratory. SeL17 = leaves of *G. heterophylla* from 2017 collection. SeS17 = stems of *G. heterophylla* from 2017 collection. SeR17 = roots of *G. heterophylla* from 2017 collection. SeL18 = leaves of *G. heterophylla* from 2018 collection. SeS18 = stems of *G. heterophylla* from 2018 collection. SeR18 = roots of *G. heterophylla* from 2018 collection.

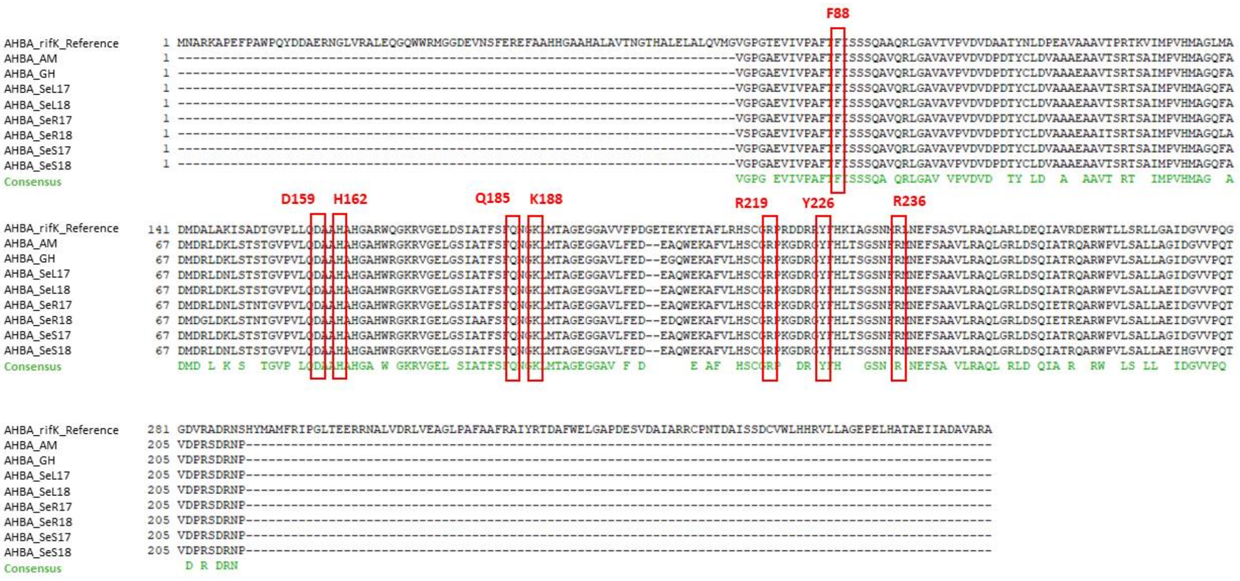

Figure S2. Multiple amino acid alignments of the translated AHBA synthase gene obtained from leaves stems, and roots of *G. heterophylla* originated from South Africa and the reference AHBA synthase enzyme encoded by the rifK gene from *Amycolatopsis mediterranei*. The highlighted amino acids in red and boxes indicate conserved active residues of the AHBA synthase enzyme from *A. mediterranei*.

```
rcbL_GH        1 CAGAGACTAAAGCGAGTGTTGGATTCAAGGCTGGCGTTAAAGATTATAAATTGACTTATTATACTCCTGACTATGAAACCAAAGATACTGATATCTTGGCAGCATTCCGAGTAACTCCTCAACCTGGAGTTCCGCCTGAA
rcbL_SeL17     1 ............................................................................................................................................
rcbL_SeL18     1 ............................................................................................................................................
rcbL_SeR17     1 ............................................................................................................................................
rcbL_SeR18     1 ............................................................................................................................................
rcbL_SeS17     1 ............................................................................................................................................
rcbL_SeS18     1 ............................................................................................................................................

rcbL_GH      141 GAAGCAGGGGCGGCGGTAGCTGCTGAATCTTCTACTGGTACATGGACAACTGTGTGGACCGATGGGCTTACCAGTCTTGATCGTTACAAAGGACGATGCTACCACATCGAGCCCGTTGCTGGAGAAGAAAGTCAATTTAT
rcbL_SeL17   141 ............................................................................................................................................
rcbL_SeL18   141 ............................................................................................................................................
rcbL_SeR17   141 ............................................................................................................................................
rcbL_SeR18   141 ............................................................................................................................................
rcbL_SeS17   141 ............................................................................................................................................
rcbL_SeS18   141 ............................................................................................................................................

rcbL_GH      281 TGCTTATGTAGCTTATCCTTTAGACCTTTTCGAAGAAGGTTCTGTTACTAACATGTTTACTTCCATTGTGGGTAATGTCTTTGGGTTCAAAGCCCTGCGCGCTCTACGTCTGGAGGATTTGCGAATCCCCCCTGCTTATT
rcbL_SeL17   281 ............................................................................................................................................
rcbL_SeL18   281 ............................................................................................................................................
rcbL_SeR17   281 ............................................................................................................................................
rcbL_SeR18   281 ............................................................................................................................................
rcbL_SeS17   281 ............................................................................................................................................
rcbL_SeS18   281 ............................................................................................................................................

rcbL_GH      421 CTAAAACTTTCCAAGGCCCGCCGCATGGTATCCAAGTTGAGAGAGATAAATTGAACAAGTATGGACGCCCTCTATTGGGGTGTACTATTAAACCTAAATTGGGATTATCCGCTAAGAATTATGGTAGAGCAGTTTATGAA
rcbL_SeL17   421 ............................................................................................................................................
rcbL_SeL18   421 ............................................................................................................................................
rcbL_SeR17   421 ............................................................................................................................................
rcbL_SeR18   421 ............................................................................................................................................
rcbL_SeS17   421 ............................................................................................................................................
rcbL_SeS18   421 ............................................................................................................................................

rcbL_GH      561 TGTCTCCG
rcbL_SeL17   561 ........
rcbL_SeL18   561 ........
rcbL_SeR17   561 ........
rcbL_SeR18   561 ........
rcbL_SeS17   561 ........
rcbL_SeS18   561 ........
```

Figure S3. A multiple alignments of amplified rcbL gene obtained from leaves stems, and roots of *G. heterophylla* originated from South Africa from 2017 and 2018 collections using global alignment from Clone Manager 9 Professional edition. The dot indicates the identical nucleotide. QH = *G. heterophylla* cultivating in our laboratory, the authentic material. SeL17 = leaves of *G. heterophylla* from 2017 collection. SeS17 = stems of *G. heterophylla* from 2017 collection. SeR17 = roots of *G. heterophylla* from 2017 collection. SeL18 = leaves of *G. heterophylla* from 2018 collection. SeS18 = stems of *G. heterophylla* from 2018 collection. SeR18 = roots of *G. heterophylla* from 2018 collection.

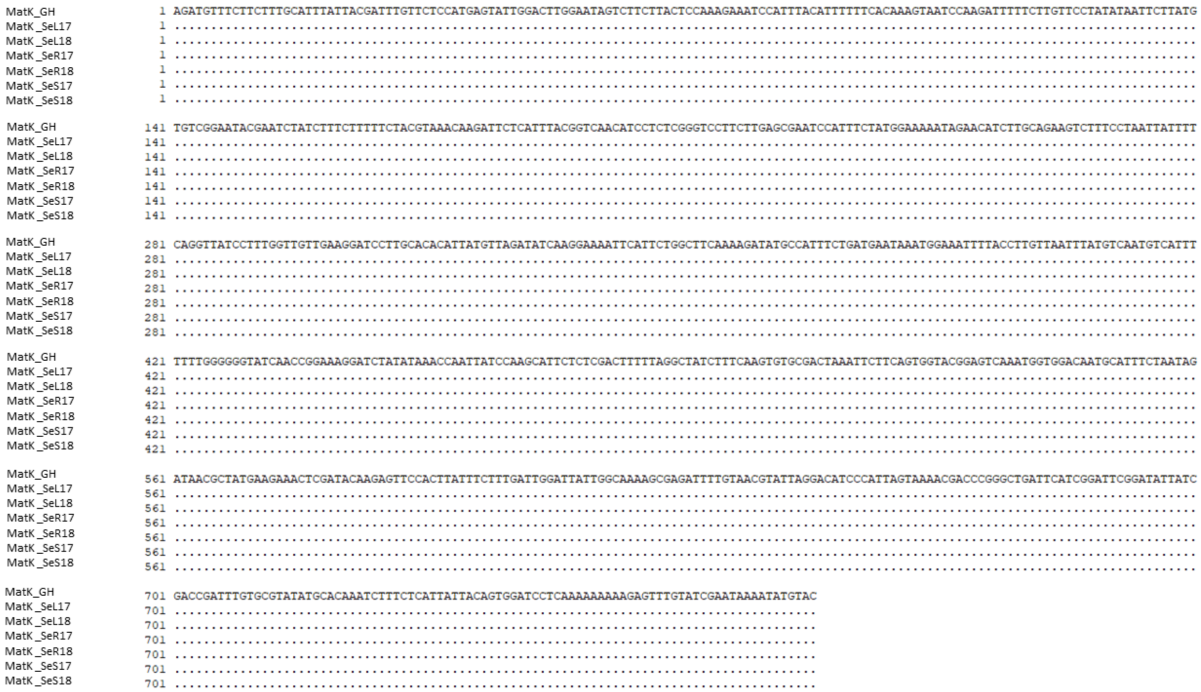

Figure S4. A multiple alignments of amplified matK gene obtained from leaves stems, and roots of *G. heterophylla* originated from South Africa from 2017 and 2018 collections using global alignment from Clone Manager 9 Professional edition. The dot indicates the identical nucleotide. QH = *G. heterophylla* cultivating in our laboratory, the authentic material. SeL17 = leaves of *G. heterophylla* from 2017 collection. SeS17 = stems of *G. heterophylla* from 2017 collection. SeR17 = roots of *G. heterophylla* from 2017 collection. SeL18 = leaves of *G. heterophylla* from 2018 collection. SeS18 = stems of *G. heterophylla* from 2018 collection. SeR18 = roots of *G. heterophylla* from 2018 collection.

Table S1. Overview of the halogenase gene received from *A. mirum* (our reference) and presented in the roots of *G. heterophylla* collected from South Africa.

Name of the samples	Sequence length (bp)	Accession number	Microorganism	GenBank ID	Sequence ID	% homology
Reference						
Am_Halogenase	454	OL944594	*Actinosynnema mirum* DSM 43827	ACU37095.1	CP001630.1	100%
2017 collection						
2017-SeR-Halogenase-1	449	OK754597	*Catenulispora acidiphila* DSM 44928	ACU69473.1	CP001700.1	78%
2017-SeR-Halogenase-2	512	OK649330	*Amycolatopsis sp*. WAC1375	AGE12641.1	JX576190.1	85%
2017-SeR-Halogenase-3	462	OK649333	*Variovorax sp*. PAMC28562	QNK76028.1	CP060296.1	80%
2017-SeR-Halogenase-4	476	OK754598	*Amycolatopsis sp*. YIM 10	QFU88391.1	CP045480.1	81%
2017-SeR-Halogenase-5	494	OK649331	*Streptomyces chartreusis* ATCC 14922	QEV72535.1	CP023689.1	78%
2018 collection						
2018-SeR-Halogenase-1	456	OK754599	*Urbifossiella limnaea* ETA A1	QDU18131.1	CP036273.1	80%
2018-SeR-Halogenase-2	474	OK649332	*Amycolatopsis sp*. WAC1375	AGE12641.1	JX576190.1	86%
2018-SeR-Halogenase-3	457	OK649335	*Catenulispora acidiphila* DSM 44928	ACU69473.1	CP001700.1	78%
2018-SeR-Halogenase-4	443	OK649334	*Variovorax sp*. PAMC28562	QNK76028.1	CP060296.1	80%
2018-SeR-Halogenase-5	456	OK649336	*Catenulispora acidiphila* DSM 44928	ACU69473.1	CP001700.1	78%
2018-SeR-Halogenase-6	458	OK754600	*Amycolatopsis sp*. YIM 10	QFU88391.1	CP045480.1	82%

Table S2. Obtained halogenase gene sequences from *A. mirum* (our reference) and the roots of *G. heterophylla* collected from South Africa.

Name of the samples	Sequence length (bp)	Accession number	Sequences
Reference			
Am_Halogenase	454	OL944594	GTGCGGGAGGGCGCGCAGGTCGTCGGGCTGCTGGAGGAGGGCGGCCGGG TGGTGGGCGTGCGCTACCGCGAGGGCGGGTCGAGCGCGAGGAGCGGGC CCGGTACGTGGTGGACGCCACCGGCCGGGCAGGCGTGGTCGCCAACCGGT TCGGGCTGCGGCGGATGATCGAGGACCTGCGCATGGTGGCGGTGTTCCAC CACCGCGACGGGCTGGACGAGGCGCACAACCCCGGCCACGAGGGCGACA TCCAGGTCGGCAGTCACGCGGACGGGTGGATCTGGGCGATCCCGCTGTCG GCGGACCGGATCAGCGTGGGCACGGTGATGCACCGCGACCGGCTGCGCG GTCGCACCCCCGCCGAGGCGTTCGCCGAGCACGTCGAGCGGGTGCCCAGG ATCAACCAGCGGCTCACCGGCACCACGGCGACCTCGGACTTCTGGGTGGA GACCzGACA
2017 collection			
2017_SeR_Halogenase_1	449	OK754597	TCCTCGACCTGTTGGGCGCCCGCGAAACGGTGGCGAATCACGGCTTCGTG CGCAAGGACGGCGCGTACTTCGAATGGGGCCCGGAGAACTGGGACCTCG ACTTCAACCACCTGACCGGCGCCAACGGCTACAGCTACCAGGTGATCCGC TCCGAGTTCGACCACCTGTTACTCAAACGGGCCGCGGACAACGGCGTCGA TGTCCGCGAGGGGGTCAGGGTCGTGGAGCTGACCTTCGACGATGAGCGGC CGGTCGCCGCGCGCTGGGTGGCTACCGGTGACCGGGAAACCGGCGGTACG ATCGAGTTCGACTTCCTGGTCGACGCCTCTGGCCGGGCCGGCGTGATGGCC ACCAAGTACCTGCGCAACCGCACCTACCTGGAGGCGTTCAGGAACGTCGG CGTCTGGTCGTACTGGCGGGGGGTTACCCCGCTCGACCGCGGTCCGAAG

Table S2. Obtained halogenase gene sequences from *A. mirum* (our reference) and the roots of *G. heterophylla* collected from South Africa. (continue)

Name of the samples	Sequence length (bp)	Accession number	Sequences
2017 collection (continue)			
2017_SeR_Halogenase_2	512	OK649330	GCTTTCCCGCGGTACCAGATCGGGGAGTCGTTGCTGCCGTCGACGGTCCAT GGCGTGTGCCGCATGCTCGGCGCCGCGGACGAACTGGCGGAGGCGGGTTT CCCGGTGAAGCGGGGCGGCACGTTCCGCTGGGGCGCTCGTCCGGAGCCGT GGACGTTCTCCTTCTCTGTCTCCCCGAGGATCACGGGTCCGTCGTCGTTCGC CTATCAGGTGGAACGGGCGCGGTTCGACCAGATCTTGCTGAACAACGCCA AGCGCAAGGGTGTGGTGGTGCGGGAGGGGAGCACGGTCACCGGTGCGCT GGAGGAGGGTGAGCGTGTTACGGGTTTGCGTTACGCCGATCCCGACGGTA ACCAGCACGAGGTGTCCGCGACTTTCGTGATCGACGCGTCGGGCAACAAG AGTCGTCTGTACTCCAATGTCGGTGGCTCGCGGAACTATTCGGAGTTCTTC CGCAGCTTGGCGCTGTTCGGCTACTTCGAGGGCGGCAAGCGGCTGGCCGC TCCGTACTC
2017_SeR_Halogenase_3	462	OK649333	GTCGCGCTGTTCGACAGGCTCGGCGTACGCGACCAGATCGAGCGCATCGG CATGCCCAAGTTTGGCGTCGAATTCGTGTCGCCGGATCACGATCGCGCCGC TTTCGTCGAATTCGCCGAGGCCTGGGACAAGTCGATGCCCTATGCCTGGCA GGTGCGACGCTCCGAAATGGACGAGATCCTCTTTCGCAACGCCGCGGCGC GGGGCGCGGAGGCCATCGAAGGCTGCCGGGTGCGCGAAGTCAGCTTCGA CGCCGATGGTGCCCACGTGCAGGCGGTGCTCGACGATGGAACCCGGCGTC AATGGCGGGCGCGCTTCGTACTCGATGCCTCCGGCCGCGACACCTTCCTGT CGAACAAGCTCAAGGCCAAGCTGAAGAACCCGCGCCACAACAGCTCTGC GCTGTTCGGCCATTTCGTGAACGCCGAGCGACTGCCCGGCAAGCTCGAAG GCAACATCACG

Table S2. Obtained halogenase gene sequences from *A. mirum* (our reference) and the roots of *G. heterophylla* collected from South Africa. (continue)

Name of the samples	Sequence length (bp)	Accession number	Sequences
2017 collection (continue)			
2017_SeR_Halogenase_4	476	OK754598	GCACGGGATCTGCGCCATGCTCGGCGTCCGGGACGAGGTCGAGCGGGCCG<br>GGTTCGTCCGGAAACGGGGCGGCACCTTCAAATGGGGGTCGAACCCGGAC<br>CCGTGGACGTTCGCGTTCGCCGCGTCGGAGCGGATGGCGGGGCCGACGTC<br>CTACGCCTACCAGGTCGAGCGCACCCGCTTCGACGCCCTGCTGCTCGACA<br>ACGCCGTGCGGTGCGGCGCCGACGTCCGGCAGGGGCATCGCGTGCTCGGC<br>ACGCTGCGCGACGAGGACGGCCGGATCGCCGGTGCCCGCTACCTCGACGA<br>CGCGGGGCGGGAGGGGCACGCCACCGCGCGGTTCGTCGTGGACGCGTCG<br>GGCAACGGCAGCACGCTGCACAACGACATCGGCGGCGCCCGCGAGTACT<br>CACCGCATTCCCGCAACCTGGCGCTGTTCGGCTACTTCGCGGGCGGCCGCC<br>GGGCACCGGAACCCAACGCGGGCAACA
2017_SeR_Halogenase_5	494	OK649331	CACGGCCTGTGCCGGCTGCTCGGCGTGGCCGACGAGGTGGCCGGCGCCGG<br>GTTCACCCTCAAACGCGGCGGCACGTTCCGCTGGGGCCGTAGCGCAGAGC<br>CCTGGCAGTTCAGCTTCGCCATGTCGCCCCGGGTGCCGGAGCCGATGTCCA<br>CCGCGTTCCAGGTCGAGCGGTCCCGGTTCGACGAGATCCTGCTGCGCAAC<br>GCCGCCGACAACGGCGTGGTCGTCCGAGAGGGCAACCGGGTGCGCGCGG<br>TCACCCGGGACGGCACCGGCCGGGTCGACGGGGTCGAGTACACCGGCCG<br>GGACGGCGTGGCGCACACCGTGGCCGCCCGCTACGTGGTGGACGCGTCGG<br>GCAACACCAGCAGGATCCACGGTCAGGTCGGCGCCAGGGTGCGGTCCGA<br>CTTCTTCCGCGACCTCGCGGTGTTCGGCTACTTCGCCGGCGGTGCCCGGCT<br>GCCGCGACCGCACAGCGGCAACATCTTCTGCGCCACCTTCGACGC

Table S2. Obtained halogenase gene sequences from *A. mirum* (our reference) and the roots of *G. heterophylla* collected from South Africa. (continue)

Name of the samples	Sequence length (bp)	Accession number	Sequences
2018 collection			
2018_SeR_Halogenase_1	456	OK754599	AGCGGCTCGGCATGCTCGACAAGATGAAGGGGAGCCACTTCGTCAAGAA<br>GTACAGCGTCCAGTTCGTCAACCAGCACGGCAGGCTGTCCGAGCCGTTCT<br>ATTTCCTCGACCACCGGCCGCACGAGTCGTCGCAGACGTGGCAGGTCAAG<br>CGCGAGGAGCTTGACGTGGCGCTGCTCGACAACGCCCGCGAGCACGGCGC<br>CGAGGTGCATGAAGGCGTGCGCGTCCTGAACGTGCTGTTCGAGGGGGACC<br>GGGCCGTCGGCGTCCGCATCCAGGACGAGGCGGGGGCCGAGCGGGAGGT<br>GCGGGCGAAGGTCATCGTGGACGCGAGCGGGCAGGGGTCGCTGATCATG<br>AGCCGGCTCGGCCTGCGCGAGTGGGACCCGGTCCTCAAGAAGGCCGCCCT<br>GTGGACGTACTGGGAGGGCGCCTACCGCGACACCGGCAAGGACGAGGGG<br>GCGACGCTCG
2018_SeR_Halogenase_2	474	OK649332	CATGGCGTGTGCCGCATGCTCGGCGCCGCGGACGAACTGGCGGAGGCGGG<br>TTTCCCGGTGAAGCGGGGCGGCACGTTCCGCTGGGGCGCTCGTCCGGAGC<br>CGTGGACGTTCTCCTTCTCTGTCTCCCCGAGGATCACGGGTCCGTCGTCGTT<br>CGCCTATCAGGTGGAACGGGCGCGGTTCGACCAGATCTTGCTGAACAACG<br>CCAAGCGCAAGGGTGTGGTGGTGCGGGAGGGGAGCACGGTCACCGGTGC<br>GCTGGAGGAGGGTGAGCGTGTTACGGGTTTGCGTTACGCCGATCCCGACG<br>GTAACCAGCACGAGGTGTCCGCGACTTTCGTGATCGACGCGTCGGGCAAC<br>AAGAGTCGTCTGTACTCCAATGTCGGTGGCTCGCGGAACTATTCGGAGTTC<br>TTCCGCAGCTTGGCGCTGTTCGGCTACTTCGAGGGCGGCAAGCGGCTGGCC<br>GCTCCGTACTCGGGGAACATC

Table S2. Obtained halogenase gene sequences from *A. mirum* (our reference) and the roots of *G. heterophylla* collected from South Africa. (continue)

Name of the samples	Sequence length (bp)	Accession number	Sequences
2018 collection (continue)			
2018_SeR_Halogenase_3	457	OK649335	CCTCGACCTGTTGGGCACCCGCGAAACGGTGGCGAATCACGGCTTCGTGCGCAAGGACGGCGCGTACTTCGAATGGGGCCCGGAGAACTGGGACCTCGACTTCAACCACCTGACCGGCGCCAACGGCTACAGCTACCAGGTGATCCGCTCCGAGTTCGACCACCTGTTACTCAAACAGGCCGCGGACAACGGCGTCGATGTCCGCGAGGGGGTCAGGGTCGTGGAGCTGACCTTCGACGATGAGCGGCCGGTCGCCGCGCGCTGGGTGACTACCGGTGACCGGGAAACCGGCGGTACGATCGAGTTCGACTTCCTGGTCGACGCCTCTGGCCGGGCCGGCGTGATGGCCACCAAGTACCTGCGCAACCGCACCTACCTGGAGGCGTTCAGGAACGTCGGCGTCTGGTCGTACTGGCGGGGGGTTACCCCGCTCGACCGCGGTCCGAAGGGCGCCATC
2018_SeR_Halogenase_4	443	OK649334	GTTCGACAGGCTCGGCGTACGCGACCAGATCGAGCGCATCGGCATGCCCAAGTTCGGCGTCGAATTCGTGTCGCCGGATCACGATCACGCCGCTTTCGTCGAATTCGCCGAGGCCTGGGACAAGTCGATGCCCTATGCCTGGCAGGTGCGACGCTCCGAAATGGATGAGATCCTTTTTCGCAACGCCGCGGCGCGGGGCGCGGAGGCCATCGAAGGCTGCCGGGTGCGCGAAGTCAGCTTCGACGCCGACGGTGCCCACGTGCAGGCGGTGCTCGACGATGGAACCCGGCGTCAATGGCGGGCGCGCTTCGTACTCGATGCCCCCGGCCGCGACACCTTCCTGTCGAACAAGCTCAAGGCCAAGCTGAAGAACCCGCGCCACAACAGCTCTGCGCTGTTCGGCCATTTCGTGAACGCCGAGCGACTGCCCGGCAAGCTCGAAG

Table S2. Obtained halogenase gene sequences from *A. mirum* (our reference) and the roots of *G. heterophylla* collected from South Africa. (continue)

Name of the samples	Sequence length (bp)	Accession number	Sequences
2018 collection (continue)			
2018_SeR_Halogenase_5	456	OK649336	ATCCTCGACCTGTTGGGCGCCCGCGAAACGGTGGCGAATCACGGCTTCGT GCGCAAGGACGGCGCGTACTTCGAATGGGGCCCGGAGAACTGGGACCTC GACTTCAACCACCTGACCGGCGCCAACGGCTACAGCTACCAGGTGATCCG CTCCGAGTTCGACCACCTGTTACTCAAACAGGCCGCGGACAACGGCGTCG ATGTCCGCGAGGGGGTCTGGGTCGTGGAGCTGACCTTCGACGATGAGCGG CCGGTCGCCGCGCGCTGGGTGGCTACCGGTGACCGGGAAACCGGCGGTAC GATCGAGTTCGACTTCCTGGTCGACGCCTCTGGCCGGGCCGGCGTGATGGC CACCAAGTACCTGCGCAACCGCACCTACCTGGAGGCGTTCAGGAACGTCG GCGTCTGGTCGTACTGGCGGGGGGTTACCCTGCTCGACCGCGGTCCGAAG GGCGCC
2018_SeR_Halogenase_6	458	OK754600	GCACGGGATCTGCGCCATGCTCGGCGTCCGGGACGAGGTCGAGCGGGCCG GGTTCGTCCGGAAACGGGGCGGCACCTTCAAATGGGGGTCGAACCCGGAC CCGTGGACGTTCGCGTTCGCCGCGTCGGAGCGGATGGCGGGGCCGACGTC CTACGCCTACCAGGTCGAGCGCACCCGCTTCGACGCCCTGCTGCTCGACA ACGCCGTGCGGTGCGGCGCCGACGTCCGGCAGGGGCATCGCGTGCTCGGC ACGCTGCGCGACGAGGACGGCCGGATCGCCGGTGCCCGCTACCTCGACGA CGCGGGGCGGGAGGGGCACGCCACCGCGCGGTTCGTCGTGGACGCGTCG GGCAACGGCAGCACGCTGCACAACGACATCGGCGGCGCCCGCGAGTACT CACCGCATTCCCGCAACCTGGCGCTGTTCGGCTACTTCGCGGGCGGCCGCC GGGCACCGG

Table S3. Statistic analysis of the AHBA synthase gene expression of *G. heterophylla* collected from South Africa in 2017 and 2018 and cultivated at our laboratory in Germany.

		**2017-SeL**	**2017-SeS**	**2017-SeR**	**2018-SeL**	**2018-SeS**	**2018-SeR**	**GH-Lab**
2017-SeL	Mean difference	—	-0.0278	0.0102	0.01313	0.01437	0.00537	-0.02963
	p-value	—	0.083	0.004	0.004	0.002	0.867	0.131
2017-SeS	Mean difference		—	0.0380	0.04093	0.04217	0.03317	-0.00183
	p-value		—	0.044	0.041	0.038	0.041	1.000
2017-SeR	Mean difference			—	0.00293	0.00417	-0.00483	-0.03983
	p-value			—	0.194	0.080	0.904	0.074
2018-SeL	Mean difference				—	0.00123	-0.00777	-0.04277
	p-value				—	0.333	0.674	0.068
2018-SeS	Mean difference					—	-0.00900	-0.04400
	p-value					—	0.583	0.064
2018-SeR	Mean difference						—	-0.03500
	p-value						—	0.062
GH-Lab	Mean difference							—
	p-value							—

One-way ANOVA analysis with Games-Howell Post-Hoc test. GH-Lab indicated *G .heterophylla* cultivated in our laboratory. 2017-SeL, 2017-SeS, and 2017-SeR indicate the leaves, stems, and roots parts of the 2017 collection of *G. heterophylla* originated from Seweweekspoort, South Africa, while 2018-SeL, 2018-SeS, and 2018-SeR indicate the counterpart from the 2018 collection

Table S4. Statistic analysis of the FRS gene expression of *G. heterophylla* collected from South Africa in 2017 and 2018 and cultivated at our laboratory in Germany.

		2017-SeL	2017-SeS	2017-SeR	2018-SeL	2018-SeS	2018-SeR	GH-Lab
2017-SeL	Mean difference	—	0.105	0.00440	0.10897	0.1513	-0.0632	-0.00240
	p-value	—	0.005	1.000	0.002	< .001	0.247	0.999
2017-SeS	Mean difference		—	-0.10033	0.00423	0.0466	-0.1680	-0.10713
	p-value		—	0.042	0.992	0.011	0.041	< .001
2017-SeR	Mean difference			—	0.10457	0.1469	-0.0676	-0.00680
	p-value			—	0.017	0.012	0.215	0.989
2018-SeL	Mean difference				—	0.0424	-0.1722	-0.11137
	p-value				—	0.065	0.024	0.014
2018-SeS	Mean difference					—	-0.2146	-0.15373
	p-value					—	0.021	< .001
2018-SeR	Mean difference						—	0.06083
	p-value						—	0.276
GH-Lab	Mean difference							—
	p-value							—

One-way ANOVA analysis with Games-Howell Post-Hoc test. GH-Lab indicated *G .heterophylla* cultivated in our laboratory. 2017-SeL, 2017-SeS, and 2017-SeR indicate the leaves, stems, and roots parts of the 2017 collection of *G. heterophylla* originated from Seweweekspoort, South Africa, while 2018-SeL, 2018-SeS, and 2018-SeR indicate the counterpart from the 2018 collection
